# Phosphate Starvation by Energy Metabolism Disturbance in *Candida albicans* *vip1*Δ/Δ Induces Lipid Droplet Accumulation and Cell Membrane Damage

**DOI:** 10.3390/molecules27030686

**Published:** 2022-01-21

**Authors:** Xueling Peng, Congcong Ma, Yuxin Feng, Biao Zhang, Mengsen Zhu, Tianyu Ma, Qilin Yu, Mingchun Li

**Affiliations:** 1 Key Laboratory of Molecular Microbiology and Technology of the Ministry of Education, Department of Microbiology, College of Life Sciences, Nankai University, Tianjin 300071, China; pengxueling1992@163.com (X.P.); mcmacongcong@163.com (C.M.); zmshome@163.com (M.Z.); matianyu3317658@163.com (T.M.); yuqilin7007@163.com (Q.Y.); 2 School of Food and Biology Engineering, Xuzhou University of Technology, Xuzhou 221000, China; 3 State Key Laboratory of Component-based Chinese Medicine, Tianjin University of Traditional Chinese Medicine, Tianjin 300457, China; fengyn@live.com (Y.F.); zhangbiao6853@126.com (B.Z.)

**Keywords:** PHO pathway, phosphate stress, lipid droplet

## Abstract

Phosphorus in the form of phosphate (Pi) is an essential element for metabolic processes, including lipid metabolism. In yeast, the inositol polyphosphate kinase *vip1* mediated synthesis of inositol heptakisphosphate (IP_7_) regulates the phosphate-responsive (PHO) signaling pathway, which plays an important role in response to Pi stress. The role of *vip1* in Pi stress and lipid metabolism of *Candida albicans* has not yet been studied. We found that when *vip1*Δ/Δ was grown in glucose medium, if Pi was supplemented in the medium or mitochondrial Pi transporter was overexpressed in the strain, the lipid droplet (LD) content was reduced and membrane damage was alleviated. However, further studies showed that neither the addition of Pi nor the overexpression of the Pi transporter affected the energy balance of *vip1*Δ/Δ. In addition, the LD content of *vip1*Δ/Δ grown in Pi limitation medium PNMC was lower than that grown in SC, and the metabolic activity of *vip1*Δ/Δ grown in PNMC was also lower than that grown in SC medium. This suggests that the increase in Pi demand by a high energy metabolic rate is the cause of LD accumulation in *vip1*Δ/Δ. In addition, in the *vip1*Δ/Δ strains, the core transcription factor *PHO4* in the PHO pathway was transported to the vacuole and degraded, which reduced the pathway activity. However, this does not mean that knocking out *vip1* completely blocks the activation of the PHO pathway, because the LD content of *vip1*Δ/Δ grown in the medium with β-glycerol phosphate as the Pi source was significantly reduced. In summary, the increased Pi demand and the decreased PHO pathway activity in *vip1*Δ/Δ ultimately lead to LD accumulation and cell membrane damage.

## 1. Introduction

Pi plays a role in many biological processes, such as cell signaling, energy metabolism, and phospholipid synthesis. In particular, Pi is of great significance in the process of energy metabolism, and it plays an important role in regulating three main energy production pathways: glycolysis, the tricarboxylic acid cycle (TCA) and oxidative phosphorylation [1,2]. Pi regulates glycolytic metabolism by regulating the activities of glyceraldehyde 3-phosphate dehydrogenase, hexokinase and phosphofructokinase; it regulates the entry of glutamine into the TCA cycle by regulating the activities of glutaminase and dehydrogenase, thereby controlling TCA metabolism; when it regulates oxidative phosphorylation, it acts as a substrate molecule in the process of ATP synthesis, and it can also promote the activity of complex I in the electron transport chain and help the transfer of electrons from cytochrome b to cytochrome c [3,4]. Pi limitation affects energy balance.

Mitochondrial phosphate carrier (*PIC*) is a protein that transports Pi through the inner mitochondrial membrane into the mitochondria to provide Pi for oxidative phosphorylation [3]. *MIR1* is the yeast homolog of *PIC*; *Saccharomyces cerevisiae* with this gene knocked out was unable to grow in nonfermentable carbon source medium. *PIC2*, which encodes another mitochondrial Pi carrier in yeast and *Arabidopsis thaliana*, has 40% homology with *MIR1*, and it plays an important role in the stress response. These gene-deficient strains cannot grow in the medium that requires mitochondrial metabolism, which also proves the importance of *PIC* for oxidative phosphorylation. In addition, the important role of *PIC* in ATP production and oxidative phosphorylation has also been confirmed in mammalian cells [5].

Glycolysis, mitochondria and lipid metabolism are closely interrelated. In the lipid synthesis sample of *Rhodosporidium toruloides*, the levels of related proteins in the TCA cycle (such as Idh1, Sdh1, Sdh2 and Mdh1) are reduced, and the glycolytic metabolic rate will increase to some extent [6]. Pi not only regulates glycolysis and mitochondrial metabolism, but also controls lipid synthesis [1]. Pi limitation can promote the conversion of sugars to lipids. In the oleaginous yeast *R. toruloides*, Pi limitation will increase the synthesis of triacylglycerol (TG) and decrease the activity of the TCA cycle; conversely, reducing the TCA cycle will promote the flow of carbon metabolism to lipid synthesis [7].

Under Pi starvation conditions, the PHO pathway is the first to be induced, and the activation of this pathway will promote the expression of a series of Pi transporters and other related genes. In a Pi-rich medium, the Pho80–Pho85 complex is activated to phosphorylate *PHO4*, and then phosphorylated *PHO4* is transported out of the nucleus; in Pi stress conditions, Pho80–Pho85 kinase is inhibited, and *PHO4* accumulates in the nucleus and promotes the transcription of PHO-related genes. The Pho80–Pho85 complex is regulated by Pho81, and IP_7_ is necessary for Pho81-dependent inhibition of Pho80–Pho85; moreover, inactivation of Pho80–Pho85–Pho81 by IP_7_ appears to be specific [8]. IP_7_ in yeast, which regulates the PHO pathway, is 1-PP-IP_5_ synthesized by *vip1*, but IP_7_ synthesized by Kcs1 does not have this function [8,9]. However, in *Cryptococcus neoformans*, IP_7_ (isomer 5-PP-IP_5_) synthesized by Kcs1 can regulate the PHO pathway by binding to the SPX domain in Pho81 [10]. Therefore, whether *vip1*- or Kcs1-mediated synthesis of IP_7_ can regulate PHO pathway activity has different results in different species.

In conclusion, Pi is an important factor in the regulation of energy metabolism, and Pi stress influences the intracellular energy balance and LD accumulation. It has been confirmed that IP_7_ (isomer 1-PP-IP5) synthesized by *vip1* in *S. cerevisiae* and IP_7_ (isomer 5-PP-IP5) synthesized by Kcs1 in *C. neoformans* play a regulatory role in the PHO pathway, but further, the final result of inhibition of the PHO pathway after knockout of these genes has not been discussed in depth. Moreover, there is no relevant research on the relationship between the *C. albicans* PHO pathway and inositol polyphosphate. In this study, for the first time, we confirm the connection between *vip1* and the PHO pathway in *C. albicans*, and we further explore the correlation between energy metabolism and Pi supplementation. Pi limitation in *C. albicans vip1*Δ/Δ promotes LD accumulation and cell membrane damage. In summary, we link energy metabolism, Pi, LD and cell membranes to further reveal the physiological and biochemical effects of inositol polyphosphate, and we provide references for further revealing *C. albicans* lipid metabolism and even energy metabolism networks.

## 2. Results

### 2.1. Pi Regulation of LD Accumulation Is a Downstream Event of Energy Metabolism

**Figure 1:**
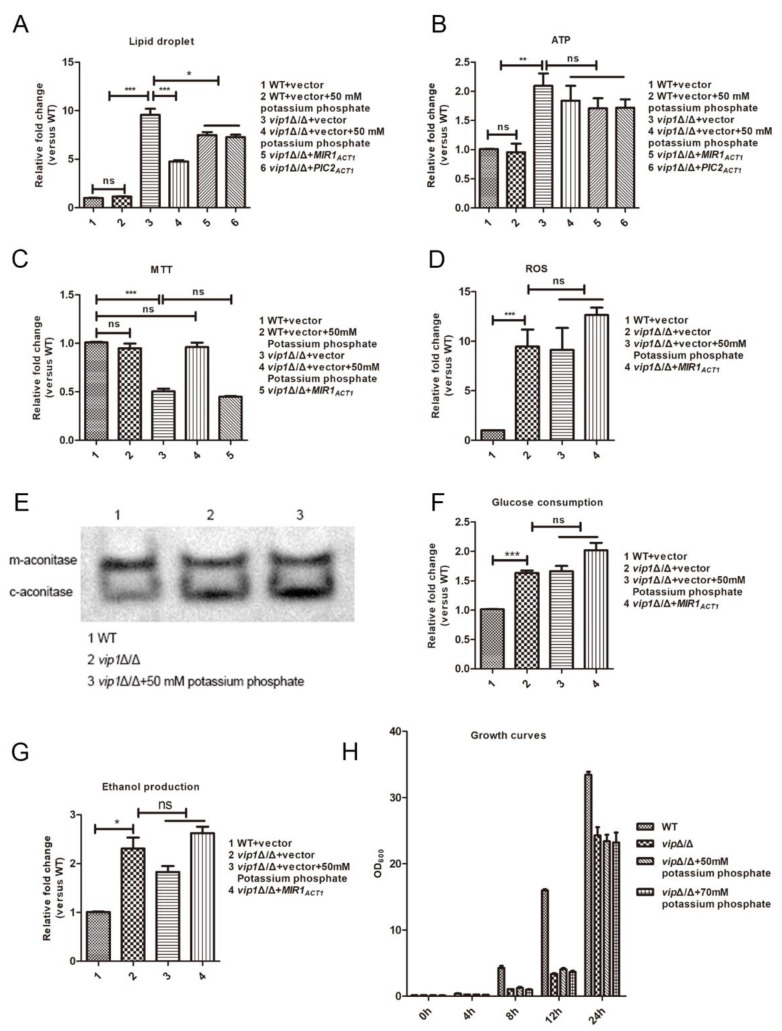
Pi cannot affect the energy balance of *vip1*Δ/Δ. (**A**) Determination of LDs. Strains were grown in SC or SC medium with Pi to stationary phase, and the cells were collected stained with Nile red, and the fluorescence intensity was measured with a microplate reader (Perkin Elmer, Waltham, MA, USA). (**B**) ATP content determination. Collected strains were grown in SC or SC medium with Pi to determine ATP content. (**C**) MTT assay for measuring mitochondrial succinate dehydrogenase activity of strains grown in SC or SC medium with Pi. (**D**) ROS determination. The strains were grown in SC or SC medium with Pi. The ROS content of the strains was stained with DCFH-DA, and the fluorescence intensity was measured with a microplate reader (Perkin Elmer, USA). (**E**) Mitochondrial aconitase activity was measured using the IGA method. The strains were grown in SC or SC medium with Pi. Strains were collected and glass beads were added, followed by vortexing to break the strain and then centrifugation to collect the supernatant for aconitase activity determination. (**F**) Glucose consumption rate. (**G**) Ethanol production. (**F**,**G**) After growing the strain in SC or SC medium with Pi, the mid-log phase strains were collected and centrifuged, and the supernatant was used for the determination of glucose and ethanol content. (**H**) Growth curve. Strains were grown in SC or SC medium supplemented with Pi, and the OD_600_ was measured at 4, 8, 12 and 24 h. * *p <* 0.05, ** *p <* 0.01, *** *p <* 0.001, ns: no significance.

*C. albicansvip1* knockout reduces the growth rate of strain in glucose medium and accumulates a large number of LDs [11]. If Pi was added to the medium, it was found that supplementation Pi could reduce the LD content in the *vip1*Δ/Δ strain (Figure 1A). This means that the Pi content in *vip1*Δ/Δ strains cannot meet the needs of cell metabolism, and Pi supplementation reduces the content of intracellular LDs. That is, LD metabolism is related to Pi in *vip1*Δ/Δ.

Pi is an important nutrient that regulates glycolysis and mitochondrial metabolism [4]. Our previous research found that the increase in the glycolytic rate of *C. albicans vip1*Δ/Δ accompanied by a decrease in mitochondrial activity was the cause of LD accumulation. Drug interference or plasmid overexpression was used to disturb energy balance to affect LD accumulation [11]. Because the content of Pi affects the energy balance, and energy metabolism disorder in *C. albicans vip1*Δ/Δ is the cause of LD accumulation, we asked, in *C. albicans vip1*Δ/Δ, whether Pi-regulated LD accumulation is achieved by affecting the energy balance.

Glycolysis is a metabolism that consumes Pi, and this metabolic pathway competitively binds to the Pi that enters the mitochondria. To this end, we sought to explore whether the decreased mitochondrial activity was due to insufficient Pi content in mitochondria. We overexpressed the two mitochondrial Pi transporters *MIR1* and *PIC2* in *vip1*Δ/Δ and determined the content of LDs in the strains at this time. The results showed that the content of LDs in the *vip1*Δ/Δ + *MIR1_ACT1_* and *vip1*Δ/Δ + *PIC2 _ACT1_* strains was reduced to a certain extent compared to that in the *vip1*Δ/Δ (Figure 1A). However, this does not mean that the increased Pi transport in mitochondria affects energy homeostasis, thus resulting in a decrease in LD content. Adding Pi to the medium, or overexpressing *MIR1* or *PIC2*, did not affect the ATP content of the *vip1*Δ/Δ strains (Figure 1B). That is, Pi content was not the factor affecting the energy balance of *vip1*Δ/Δ. Mitochondria-related metabolism, such as ROS and aconitase activity, in the *vip1*Δ/Δ strain under Pi supplementation medium was not affected, compared to that in the medium without Pi (Figure 1D,E). Moreover, we found that supplementation with Pi promoted the succinate dehydrogenase activity of *vip1*Δ/Δ, but overexpression of the mitochondrial Pi transporter did not have this effect [12,13] (Figure 1C). Glycolysis metabolism (e.g., glucose consumption and ethanol production) was not affected by Pi content, as we supplemented Pi or overexpressed the Pi transporter in *vip1*Δ/Δ (Figure 1F,G). Adding Pi did not affect the growth of *vip1*Δ/Δ (Figure 1H). These results showed that Pi was a factor that influenced the LD accumulation, but this effect was not achieved by interference with the energy homeostasis of *vip1*Δ/Δ.

**Figure 2:**
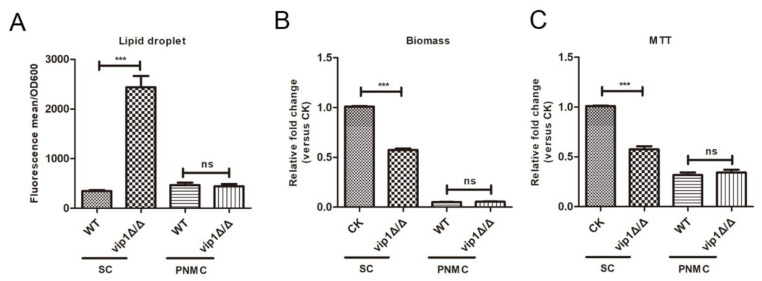
Pi regulation of LD deposition is a downstream event of energy metabolism. (**A**) Determination of LDs. The strain was grown in SC or PNMC medium to the stationary phase, the strains were collected and the LD content was measured. (**B**) Biomass determination. Strains were grown in SC or PNMC medium to the stationary phase, and biomass was measured by OD_600_. (**C**) MTT assay for measuring mitochondrial succinate dehydrogenase activity, which characterizes the metabolic activity of the strain. CK: WT strain was grown in SC medium. *** *p <* 0.001, ns: no significance.

PNMC is a Pi-limited medium [14]. If strains were grown in the PNMC, the LD content in the *vip1*Δ/Δ was not different from that in the WT (Figure 2A), which suggests that the *vip1*Δ/Δ grown in this condition had a sufficient Pi to meet the growth requirement. Comparing the growth of strain in SC or PNMC medium, we found that in the Pi limitation medium of PNMC, the biomass between the WT and *vip1*Δ/Δ strains was not significantly different, and the biomass of the strain under this condition was significantly lower than that of the strain in the SC (Figure 2B). The MTT experiment showed that the growth activity of the WT and *vip1*Δ/Δ strains in the PNMC was significantly lower than that of the strain in SC medium (Figure 2C). This means that Pi starvation is a downstream event of energy disorders or that energy metabolism disorders lead to Pi stress.

Pi is an important element in the process of energy metabolism, but the cause of *C. albicans vip1*Δ/Δ energy metabolism disorder is not Pi limitation. In summary, *C. albicans vip1*Δ/Δ energy metabolism disorder is an upstream factor for Pi stress.

### 2.2. Decreased Activity of PHO Pathway in vip1Δ/Δ Strain

The PHO signaling network is an important regulatory pathway for Pi sensing and acquisition [15], and it responds to Pi stress by regulating the activity and localization of the transcription factor *PHO4* [1,16]. Pho84 is a high-affinity Pi transporter for transporting extracellular Pi into the cytoplasm during Pi starvation, and its transcription is regulated by *PHO4* in the PHO pathway [17,18,19].

**Figure 3:**
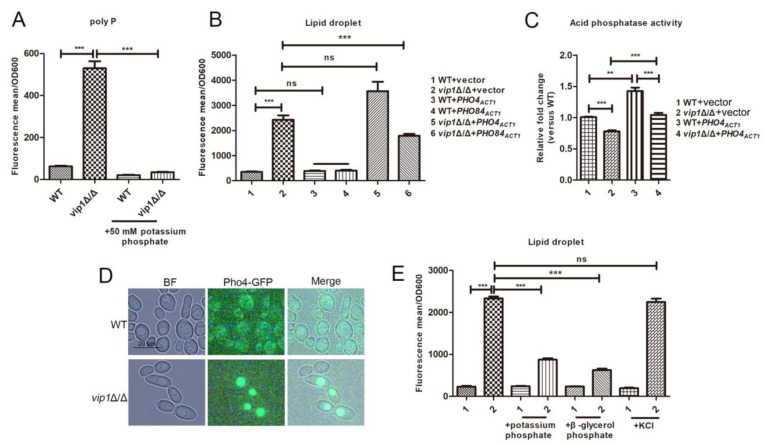
Knockout of *vip1* in *C. albicans* results in decreased activity of the PHO pathway. (**A**) polyP content determination. The strain was grown in SC or SC medium supplemented with Pi, and the polyP content was determined. (**B**) LD content determination. (**C**) Acid phosphatase activity determination. (**D**) The WT-Pho4-GFP and *vip1*Δ/Δ-Pho4-GFP strains were grown in SC medium for 4–6 h, and the location of Pho4-GFP was observed by fluorescence microscopy (BX-53, Olympus, Japan). (**E**) Determining the LD content of *vip1*Δ/Δ when grown in SC or SC medium supplemented with Pi, β-glycerol phosphate or KCl. 1: WT, 2: *vip1*Δ/Δ. ** *p <* 0.01, *** *p <* 0.001, ns: no significance.

To a certain extent, the content of polyP can characterize whether the strain is in a Pi starvation condition. We found that the polyP content in *vip1*Δ/Δ was significantly higher than that in WT, and after the addition of Pi, the content of polyP in *vip1*Δ/Δ was significantly reduced (Figure 3A). This phenomenon indicates that the intracellular Pi of *vip1*Δ/Δ is insufficient to meet the growth requirement [20]. At this time, overexpression of *PHO4* increased the activity of the PHO pathway, thus alleviating Pi stress [21], but the results showed that the LD content in *vip1*Δ/Δ + *PHO4_ACT1_* was not significantly different from that in *vip1*Δ/Δ. However, overexpression of *PHO84*, a downstream gene in the PHO pathway, mediated its expression by *PHO4*, and the LD content in *vip1*Δ/Δ+*PHO84_ACT1_* was significantly lower than that in *vip1*Δ/Δ (Figure 3B). Acid phosphatase activity was used as the readout for *PHO4* activation, and *PHO4* activation characterizes the activation of the PHO pathway [18]. The acid phosphatase results demonstrated that *vip1* gene knockout reduced the activity of the PHO pathway, which then reduced Pi acquisition by *vip1*Δ/Δ (Figure 3C). Moreover, even though the PHO pathway activity of the WT + *PHO4_ACT1_* or *vip1*Δ/Δ + *PHO4_ACT1_* strains was upregulated, the LD content in *vip1*Δ/Δ + *PHO4_ACT1_* was no different from that in *vip1*Δ/Δ (Figure 3B,C), which suggests that although overexpression of *PHO4* helps to promote PHO pathway activation and increase acid phosphatase activity, thereby helping cells acquire Pi [21], this still cannot meet the requirement of Pi for *vip1*Δ/Δ [18]. Overexpression of *PHO84*, a Pi transporter, helps *vip1*Δ/Δ acquire Pi. At this time, the content of LDs in *vip1*Δ/Δ was reduced. In addition, by observing the location of *PHO4* in WT and *vip1*Δ/Δ, it was found that the *PHO4* in *vip1*Δ/Δ in the mid-log phase was transported to the vacuole for degradation (Figure 3D), indicating that the PHO pathway in this strain was hindered. However, when β-glycerol phosphate was used as the source of Pi, the LD content in *vip1*Δ/Δ was significantly reduced (Figure 3E). Because Pi from β-glycerol phosphate needs to be metabolized by acid phosphatase synthesized by the PHO pathway [21], *vip1*Δ/Δ can mobilize Pi from β-glycerol phosphate, indicating that the PHO pathway is activated in this environment. At the same time, we used KCl as a control. We found that there was no effect on the LD content of *vip1*Δ/Δ when grown in KCl, thus clarifying the regulation of Pi on the LD of *vip1*Δ/Δ (Figure 3E). From the above results, we speculate that compared with WT, the PHO pathway in *vip1*Δ/Δ may require more stimulation to be activated but not completely blocked. This is consistent with the results in *S. cerevisiae* that under Pi limitation, *vip1*Δ/Δ strains need to be longer than WT to activate the PHO pathway [22].

In *C. albicans*, knocking out *vip1* reduces the activity of the PHO pathway and therefore leads to partial accumulation of LDs.

### 2.3. Pi Supplementation Can Improve the Cell Membrane Properties of vip1Δ/Δ

In *C. albicans vip1*Δ/Δ, the addition of Pi not only reduced LD accumulation, but also improved cell membrane properties. This means that *vip1* knockout leads to insufficient Pi in the strain to meet the metabolic requirement.

**Figure 4:**
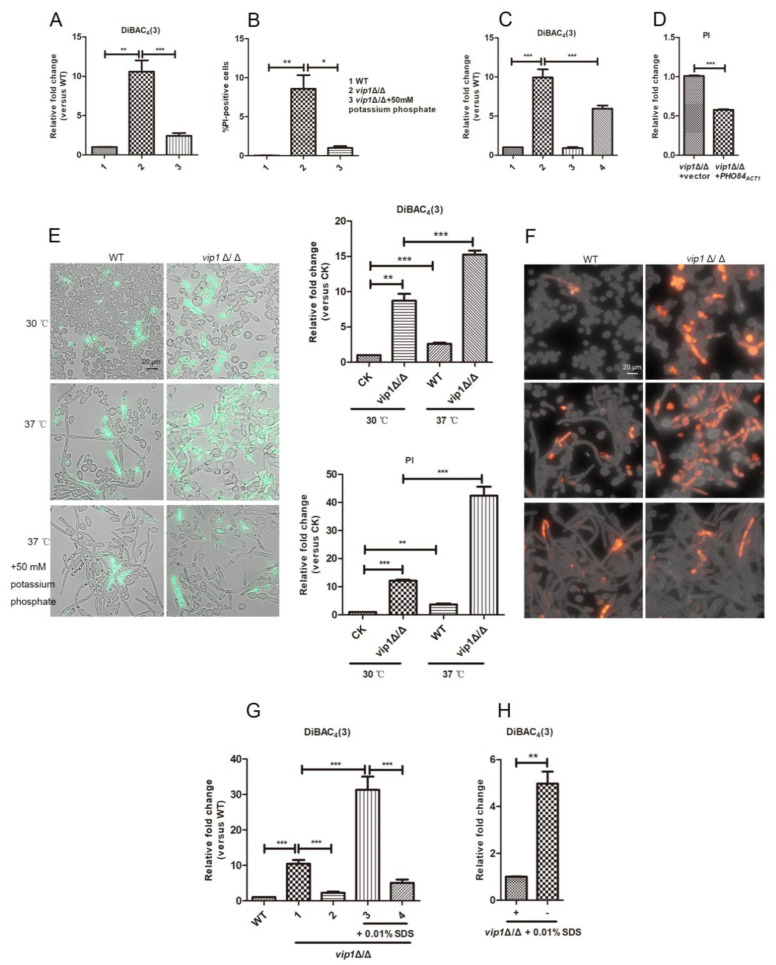
The addition of Pi helps to alleviate the membrane damage of *vip1*Δ/Δ. Adding Pi can reduce the membrane potentials (**A**) and permeabilities (**B**) of *vip1*Δ/Δ. (**A**,**B**) 1 and 2: WT or *vip1*Δ/Δ strains grown in SC, 3: *vip1*Δ/Δ strains grown in SC supplemented with Pi. (**C**) Overexpression of *PHO84* reduced the membrane potential of *vip1*Δ/Δ. 1–4: WT+vector, *vip1*Δ/Δ+vector, WT+*PHO84_ACT1_*, *vip1*Δ/Δ+*PHO84_ACT1_*. (**D**) Overexpression of *PHO84* can reduce the membrane permeabilities of *vip1*Δ/Δ. The addition of Pi can reduce the membrane potentials (**E**) and permeabilities (**F**) of *vip1*Δ/Δ under stimulation at 37 °C. (**E**,**F**) The strain was grown in SC or SC medium supplemented with Pi at 30 or 37 °C. Cell staining was performed, and the fluorescence was observed with a microscope or the fluorescence intensity was measured with a microplate reader. CK: WT strains grown in SC at 30 °C. (**G**) Pi supplementation reduced the degree of membrane damage in *vip1*Δ/Δ strains when stimulated by SDS. WT: WT was grown in SC for 2 days; 1: *vip1*Δ/Δ was grown in SC for 2 days; 2: *vip1*Δ/Δ was grown in SC with Pi for 2 days; 3: *vip1*Δ/Δ was grown in SC for 1 day, then treated with 0.01% SDS and allowed to grow for 1 day; 4: *vip1*Δ/Δ was grown in SC with Pi for 1 day, then treated with 0.01% SDS and allowed to grow for 1 day. The strain was collected, and DiBAC_4_(3) staining was used to measure the membrane potentials. (**H**) The *vip1*Δ/Δ strain that grew to the stationary phase was treated with Pi, and the strain’s resistance to SDS was enhanced. +: *vip1*Δ/Δ was grown in SC for 1 day, treated with Pi directly and allowed to grow for 1 day. Then, 0.01% SDS was added for another day, and the strains were collected to measure the cell membrane potential. -: *vip1*Δ/Δ was grown in SC for 2 days, treated with 0.01% SDS, grown for another day and collected to determine the cell membrane potential. (**A**–**H**) The strain fluorescence intensity was measured with a microplate reader (Perkin Elmer, USA). * *p <* 0.05, ** *p <* 0.01, *** *p <* 0.001.

Adding Pi to the medium or overexpressing the Pi transporter *PHO84* in the *vip1*Δ/Δ strain improved the cell membrane potential and permeability (Figure 4A–D). This result confirmed the effect of Pi on the cell membrane properties of *vip1*Δ/Δ.

Moreover, we found that Pi also has an effect on the strain under damage to the membrane by stimulation. By comparing the cell membrane properties of the strains at 30 and 37 °C, it was found that the cell membrane potentials and permeabilities of *vip1*Δ/Δ at 37 °C stimulation were increased. Once Pi was added to the medium, the membrane properties of the strain were improved (Figure 4E,F).

SDS is a cell membrane damage agent [23]. *vip1*Δ/Δ was grown for one day to promote LD accumulation and then treated with 0.01% SDS. At this time, the cell membrane was further damaged compared to that of *vip1*Δ/Δ without SDS treatment; however, when *vip1*Δ/Δ was grown in a medium supplemented with Pi, *vip1*Δ/Δ by SDS treatment had significantly lower cell membrane potential changes than the strain without Pi (Figure 4G). In addition, the *vip1*Δ/Δ strain was first grown for one day to promote LD accumulation, and then Pi was added to the nutrient-exhausted medium, allowed to grow for one day and then treated with 0.01% SDS. The changes in the cell membrane potentials of *vip1*Δ/Δ under this treatment were still significantly lower than those of the strain without Pi (Figure 4H). This shows that even if the cell membrane of *vip1*Δ/Δ is damaged, the properties of the cell membrane can still be restored after Pi is supplemented. Namely, the amount of Pi affects the cell membrane properties of *vip1*Δ/Δ. Pi stress leads to membrane damage in the *vip1*Δ/Δ strain, and the strain is sensitive to heat and SDS stimulation; adding Pi partially restores the cell membrane properties and increases the resistance to external stimuli.

## 3. Discussion

### 3.1. Pi Stress by Energy Disorder Induces LD Accumulation

Concerning the regulatory role of inositol pyrophosphates in energy metabolism, it has been shown in *S. cerevisiae* that both the inositol polyphosphate kinases Kcs1 and *vip1* mediate IP_7_ synthesis, but only *KCS1* knockout affects the intracellular glycolytic metabolism and causes mitochondrial damage. The mechanism is that Kcs1-mediated synthesis of IP_7_ can pyrophosphorylate Gcr1, thereby weakening the interaction between Rap1/Gcr1 and Gcr2 and reducing the transcription of glycolysis-related genes. Therefore, *KCS1* knockout in *S. cerevisiae* leads to an increased glycolysis rate [24].

*C. albicans KCS1* knockout does not affect the energy metabolism of the strain, while *vip1* knockout results in increased glycolysis; the mitochondria of the strain are not damaged by *vip1* knockout, but when the strain is grown in glucose medium, increased glycolysis with reduced mitochondrial activity is the cause of LD accumulation [11]. The high glycolysis rate of *C. albicans* in glucose medium caused by *vip1* knockout is the reason for the energy imbalance. In this study, we found that a high metabolic rate will increase Pi consumption, thus leading to Pi starvation. However, conversely, Pi restriction is not the reason for the energy disorder of *vip1*Δ/Δ.

Pi is an important element in energy metabolism, including glycolysis, and mitochondria and lipid metabolism are all affected by Pi [3,4]. However, in our experiments, we found that Pi stress in *C. albicans vip1*Δ/Δ strains is not the cause of energy metabolism disorders. The addition of Pi or overexpression of the Pi transporter did not affect the energy balance of the *vip1*Δ/Δ strains, including ATP content, and glycolytic metabolism rate. In this regard, we speculate that Pi regulation of LDs is independent of energy metabolism.

In addition, the LD content results when the strain was grown in PNMC proved that energy metabolism disturbance is an upstream factor in the increase in Pi demand by *vip1*Δ/Δ. The results showed that there was no difference in LD content between WT and *vip1*Δ/Δ strains grown in PNMC medium, and the amounts of LDs in *vip1*Δ/Δ grown in this environment were lower than those observed when this strain was grown in SC. Furthermore, comparing the growth status of the two strains showed that the biomass and mitochondrial activity of the WT and *vip1*Δ/Δ strains in PNMC were significantly lower than those of the two strains when they were grown in SC, indicating that the growth activity and the demand for Pi were decreased in the strains grown in a nutrient-limited medium such as PNMC. This should be the reason why the LD content of *vip1*Δ/Δ grown in PNMC medium is lower than that of this strain grown in SC.

In summary, *vip1* knockout leads to an increase in Pi consumption during energy metabolism, thus resulting in Pi stress and ultimately promoting LD accumulation.

### 3.2. VIP1 Knockout Reduces the Activity of the PHO Pathway

There are different conclusions about whether IP_7_ synthesized by Kcs1 or Vip1 regulates the PHO pathway in different species [8,9,10], but in *C. albicans*, the relationship between inositol pyrophosphate and the PHO pathway has not been reported.

Activation of the PHO pathway requires the *PHO4* transcription factor to enter the nucleus to promote the expression of phosphorus-responsive genes, but we found that *PHO4* in *C. albicans vip1*Δ/Δ during the mid-log phase was transported to the vacuole to be degraded (Figure 3D). This result suggests that PHO pathway activity in *vip1*Δ/Δ is affected. The activity of acid phosphatase characterized PHO pathway activity to a certain extent, and we found that after knocking out *vip1*, the acid phosphatase activity of the strain was significantly lower than that of WT (Figure 3C). The above results demonstrate that *vip1* knockout reduces the PHO pathway activity of the strain.

After overexpression of *PHO4*, the PHO pathway activity of *vip1*Δ/Δ+*PHO4* was upregulated, but it could still not meet the strain’s demand for Pi because the LD content in *vip1*Δ/Δ+*PHO4* was not significantly different from that in *vip1*Δ/Δ. Previous reports show that in *S. cerevisiae*, the knockout of *vip1* prevents IP_7_ from binding to the Pho80–Pho81–Pho85 complex. At this time, *PHO4* is phosphorylated and cannot enter the nucleus, thus mediating the expression of a series of phosphorus-related genes. We speculate that *vip1*-mediated synthesis of IP_7_ in *C. albicans* may also have a similar mechanism. Therefore, even if *PHO4* was overexpressed, it still could not meet the Pi requirement of *vip1*Δ/Δ, but when we overexpressed the *PHO4*-mediated synthesis of downstream phosphorus-related genes such as *PHO84*, the LD content in *vip1*Δ/Δ+*PHO84* was decreased. However, if *vip1*Δ/Δ was grown in a medium with β-glycerol phosphate as the source of Pi, the LD content in the strain could still be reduced, indicating that the PHO pathway of *vip1*Δ/Δ was not completely blocked. Because β-glycerol phosphate is a molecule that is metabolized by acid phosphatase synthesized by the PHO pathway [21], the LD content of *vip1*Δ/Δ decreased when the strain was grown in a medium supplemented with this substrate, indicating that the PHO pathway in the strain was activated at this time. This result indicated that *vip1* knockout does not completely block PHO pathway activity in *C. albicans*. Previous literature pointed out that in *S. cerevisiae*, the PHO pathway in *vip1*Δ/Δ is not completely blocked but can be activated under prolonged periods [22]. Our conclusion is consistent with this research.

### 3.3. Pi Affects Cell Membrane Properties in the vip1Δ/Δ

The addition of Pi to the medium or the overexpression of Pi transporter *PHO84* could improve the cell membrane properties of the *vip1*Δ/Δ. In addition, both thermal stimulation and SDS stress further increased cell membrane damage of *vip1*Δ/Δ. At this time, if Pi was added, the membrane damage by the stimulation could also be improved.

Regarding the phenomenon that Pi supplementation can help *vip1*Δ/Δ resist membrane damage stimulation, we propose two hypotheses: Firstly, we have previously found that membrane damage in *vip1*Δ/Δ is positively correlated with LD accumulation because the accumulation of LDs causes an increase in intracellular osmotic pressure. To prevent the cell from swelling and rupturing, an increase in chitin content promotes cell wall thickening and thus leads to membrane damage [11]. In this experiment, the content of LDs in *vip1*Δ/Δ decreased and the properties of the cell membrane improved after the addition of Pi. Is this due to the decrease in intracellular osmotic pressure caused by the decrease in LD content, which resulted in a decrease in membrane damage? Secondly, Pi is a main component of phospholipids in the cell membrane, and phospholipid degradation is a major source of Pi supply during Pi stress [25,26]. At this time, the remodeling of cell membrane components will inevitably affect the properties of the membrane. In *vip1*Δ/Δ, does the content of Pi affect the membrane properties of the strain because it interferes with the lipid composition of the membrane [27,28,29,30]? Earlier studies have shown that deletion of the gene encoding the Pi transporter affects the balance of LDs and cell membrane lipid metabolism [31]; that is, Pi affects the metabolism of LDs and membrane lipids at the same time. Does Pi also affect the conversion between lipids on membranes and lipids in LDs in *vip1*Δ/Δ? Does supplementation with Pi in *vip1*Δ/Δ strains affect phospholipid-related metabolism on the membrane and thus partially repair the membrane?

The amount of Pi available in a plant is often limited. We used *C. albicans vip1*Δ/Δ as the model to explore the relationship between Pi stress and LD metabolism, which provides a wealth of theories for biological research on *C. albicans* and provides a reference for botany research.

## 4. Materials and Methods

### 4.1. C. albicans Culture

All *C. albicans* strains used in this study are listed in Table 1. The primers used to construct plasmids and strains in this study are listed in Table 2. *C. albicans* wild-type strain is BWP17 [32]. For details about gene knockout strain construction or plasmid construction, please refer to our previous article [11,33,34].

*C. albicans* was activated overnight at 30 °C in liquid YPD (2% glucose, 1% yeast extract, 2% peptone, 80 µg/mL uridine), then transferred to SC (2% glucose, 0.67% yeast nitrogen base, 0.2% amino acid mixture) or SC with Pi (50 mM KH_2_PO_4_) [25] or PNMC (1 mM MgSO_4_, 3 g/L NaCl, 1 mM CaCl_2_, 2.5 g/L peptone) medium [14], and grown to mid-log or stationary phase.

### 4.2. MTT Assay

The strain was activated overnight and transferred to SC or Pi-supplemented SC or PNMC medium. The strains in the mid-log phase were collected, washed with PBS, resuspended in 500 µL of MTT (3-(4,5)-dimethylthiahiazo (-z-y1)-3,5-di-phenytetrazoliumromide, 100 µg/mL, diluted in different media) and then incubated at 37 °C for 1 h. The pellet was resuspended in 1 mL DMSO. The supernatant was collected by centrifugation and measured by OD_570_.

### 4.3. Nile Red Staining

LD staining was performed as described previously [35]. Strains were grown to the stationary phase and collected, washed with PBS and stained with 10 µL Nile red (1 mg/mL, dissolved in acetone) at 30 °C for 30 min. The strains were detected by a microplate assay (Ex = 488 nm, Em = 580 nm) or photographed with a fluorescence microscope (BX-53, Olympus, Japan). The results were normalized to the concentration of the cells by measuring the optical density.

### 4.4. Energy-Metabolism-Related Index Measurement

For details on energy-metabolism-related experiments, including ATP content measurement, glucose consumption, ethanol production, ROS measurement and mitochondrial aconitase in-gel enzyme activity assays, please refer to our previous experimental results [11].

### 4.5. ATP Assay

Briefly, the *C. albicans* were collected and washed 3 times with PBS, vortexed 10 times and centrifuged to remove the precipitate, and then the supernatants were collected to measure ATP using an ATP assay kit (Beyotime, Shanghai, China). All the samples were taken from triplicate independent experiments.

### 4.6. Measurement of Glucose and Ethanol Levels

The cells were cultured to the mid-log phase, collected and centrifuged at 10,000× *g* for 5 min. The supernatant was used for glucose and ethanol determination. We used the glucose oxidase method assay kit (Applygen Technologies Inc, Beijing, China) to determine medium levels of glucose. The determination of ethanol content was by reference to potassium dichromate–DNS colorimetry. The results were normalized to the concentration of the cells by measuring the optical density. All the samples were taken from triplicate independent experiments.

### 4.7. Measurement of Reactive Oxygen Species Levels

DCFH-DA (2′,7′-dichlorodihydro-fluorescein diacetate, Molecular Probes, California, CA, USA) dye was used to determine ROS levels. The cells were resuspended in PBS, and the final concentration of DCFH-DA (20 mg/mL) was incubated at 37 °C for 30 min. The fluorescence of the cells was determined by the excitation wavelength of 488 nm and the emission wavelength of 520 nm in a fluorescence plate reader. The results were normalized to the concentration of the cell by measuring the optical density. All the samples were taken from triplicate independent experiments.

### 4.8. Mitochondrial Aconitase In-Gel Enzyme Activity Assays

Protein samples were separated using a non-denaturing gel, and then the gel was incubated in a coloring solution (100 mM Tris-HCl, pH 8.0, 1 mM NADPC, 2.5 mM sodium aconitate, 5 mM MgCl2, 1.2 mM MTT, 0.3 mM phenazine methosulfate and 5 U/mL isocitrate dehydrogenase). After the color had developed, pictures were taken using a gel imager. The determination of the activity of mitochondrial aconitase was by comparing the activity of cytoplasm aconitase. All the samples were taken from triplicate independent experiments.

### 4.9. DiBAC4(3) Staining to Determine Cell Membrane Potentials

Briefly, the strains were collected, resuspended in 1 mL PBS and stained with 8 µL DiBAC_4_(3) (stock solution concentration 1 mM) for 10 min, and the fluorescence intensity was measured with a microplate reader (Ex = 488 nm, Em = 520 nm) or photographed with a fluorescence microscope (BX-53, Olympus, Japan). The results were normalized to the concentration of the cells by measuring the optical density.

### 4.10. PI Staining

The strains were collected and stained with PI (propidium iodide) for 5 min, and the fluorescence intensity was measured with a microplate reader. The results were normalized to the concentration of the cells by measuring the optical density.

### 4.11. polyP Content Measurement

The strain was collected, washed with PBS, stained with DAPI and incubated at 30 °C for 15 min. The fluorescence value was measured by a microplate reader (Ex = 420 nm, Em = 550 nm).

### 4.12. Acid Phosphatase Activity

The strain was collected, and the chromogenic substrate and detection buffer were added according to the instructions of the kit. After mixing, the mixture was incubated at 37 °C for 10 min, and the absorbance was measured at 405 nm.

## 5. Conclusions

**Figure 5:**
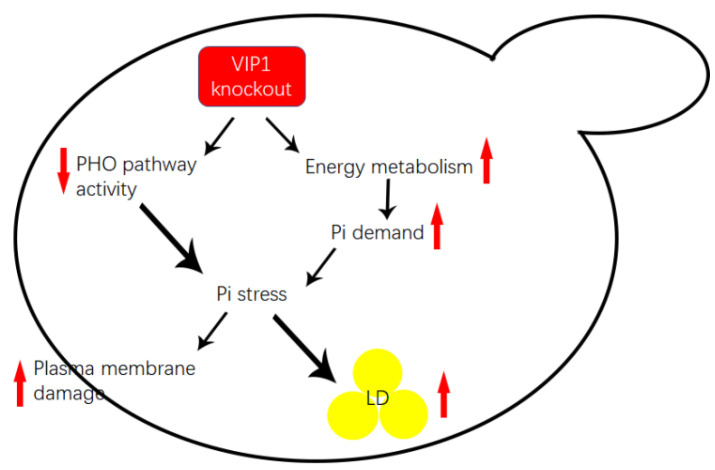
Proposed mechanisms of Pi stress in the *C. albicans vip1*Δ/Δ strain.

In this study, we found that *C. albicans vip1* knockout resulted in an increase in the glycolytic metabolism rate of the strain grown in glucose medium and thus increased the demand for Pi. The inhibition of the PHO pathway under Pi stress resulted in insufficient Pi supply in the *vip1*Δ/Δ strain, which resulted in LD accumulation and cell membrane damage. Please refer to Figure 5 for the article pattern.

## Figures and Tables

**Table 1:** Strains.

Strain	Genotype	Source
BWP17 (wild-type/WT)	*ura3Δ::λimm434/ura3Δ::λimm434 his1::hisG/his1::hisG* *arg4::hisG/arg4::hisG*	Dana A. Davis
*vip1*Δ/Δ	*ura3Δ::λimm434/ura3Δ::λimm434 his1::hisG/his1::hisG* *arg4::hisG/arg4::hisG vip1::ARG4/vip1:: dpl200*	Tianyu Ma
*vip1*Δ/Δ + *MIR1*	*ura3Δ::λimm434/ura3Δ::λimm434 his1::hisG/his1::hisG* *arg4::hisG/arg4::hisG vip1::ARG4/vip1:: dpl200,* *pACT1-MIR1*	This study
*vip1*Δ/Δ + *PIC2*	*ura3Δ::λimm434/ura3Δ::λimm434 his1::hisG/his1::hisG* *arg4::hisG/arg4::hisG vip1::ARG4/vip1:: dpl200, pACT1-PIC2*	This study
*vip1*Δ/Δ + *PHO4*	*ura3Δ::λimm434/ura3Δ::λimm434 his1::hisG/his1::hisG* *arg4::hisG/arg4::hisG vip1::ARG4/vip1:: dpl200, pACT1-PHO4*	This study
WT + *PHO4*	*ura3Δ::λimm434/ura3Δ::λimm434 his1::hisG/his1::hisG* *arg4::hisG/arg4::hisG, pACT1-PHO4*	This study
*vip1*Δ/Δ + *PHO84*	*ura3Δ::λimm434/ura3Δ::λimm434 his1::hisG/his1::hisG* *arg4::hisG/arg4::hisG vip1::ARG4/vip1:: dpl200, pACT1-PHO84*	This study
WT + *PHO84*	*ura3Δ::λimm434/ura3Δ::λimm434 his1::hisG/his1::hisG* *arg4::hisG/arg4::hisG, pACT1-PHO84*	This study
WT + Vector	*ura3Δ::λimm434/ura3Δ::λimm434 his1::hisG/his1::hisG* *arg4::hisG/arg4::hisG, pACT1*	This study
*vip1*Δ/Δ + Vector	*ura3Δ::λimm434/ura3Δ::λimm434 his1::hisG/his1::hisG* *arg4::hisG/arg4::hisG vip1::ARG4/vip1:: dpl200, pACT1*	This study
WT*-PHO4-GFP*	*ura3Δ::λimm434/ura3Δ::λimm434 his1::his G/his1::his G arg4::his G/arg4::his G* *PHO4/ PHO4-GFP::URA3*	This study
*vip1*Δ/Δ*-PHO4-GFP*	*ura3Δ::λimm434/ura3Δ::λimm434 his1::his G/his1::his G arg4::his G/arg4::his G* *vip1::CdARG4/vip1::CaHIS1* *PHO4/ PHO4-GFP::URA3*	This study

**Table 2:** Primers.

Description	Name	Sequence
*vip1* deletion, 5′ flank	*vip1*-5DR	AATTAGCAGAGACGGCGGGCCTCATTTGGATTCGCAGTTATTGGAAAGATTAAAAGAAATTTTCCCAGTCACGACGTT
*vip1* deletion, 3′ flank	*vip1*-3DR	ATCGGAAAAAATCAAGAGTATTTTAGATAACTGGGAACAATACGAATAAACTTAGAATGTTGGAATTGTGAGCGGATA
*vip1* detection, 5′ flank	*vip1*-5det	TGTCGATGGGGCAAGAATC
*vip1* detection, 3′ flank	*vip1*-3det	AAAATCGTCCGTCAAGCCT
*MIR1* plasmid construction, 5′ flank	MIR1-5′	CCGCTCGAGATGTCAACTCCAAGTGAATATA
*MIR1* plasmid construction, 3′ flank	MIR1-3′	TCCCCCGGGTTACAAAGCAACGGCTGGTGGA
*PIC2* plasmid construction, 5′ flank	PIC2-5′	CCGCTCGAGATGGCAGGAAATAGTTTAGCT
*PIC2* plasmid construction, 3′ flank	PIC2-3′	TCCCCCGGGTTAATGACCACCAGTTGTTGG
*PHO4* plasmid construction, 5′ flank	PHO4-5′	CCGCTCGAGATGGACCAGCAAGTTTGGAA
*PHO4* plasmid construction, 3′ flank	PHO4-3′	TCCCCCGGG CTTCCTTCCTTTCAACTCCT
*PHO84* plasmid construction, 5′ flank	PHO84-5′	CCGCTCGAGATGAAGTATCCCAAGGTGG
*PHO84* plasmid construction, 3′ flank	PHO84-3′	TCCCCCGGG TTAGTTTTTGACTTCTTCAGAGTC

## Data Availability

The data that support the findings of this study are available from the corresponding author upon reasonable request.

## Sample Availability

Each experiment was taken from triplicate independent experiments, and the values represent the means ± standard deviations of three experiments. The difference between the strains was compared using one-way ANOVA or one-tailed Student’s t-test. * *p* values < 0.05 were considered to be statistically significant. All statistical tests were performed using GraphPad Prism 5.
